# Use of phytopathogenic virus for peptide expression in plant system

**DOI:** 10.1186/1753-6561-8-S4-P151

**Published:** 2014-10-01

**Authors:** Francisco Jarbas Santos de Sousa, Maria Izabel Florindo Guedes, Márcia Maria Mendes Marques, Lia de Almeida Magalhães, Lucelina da Silva Araújo, Isaac Neto Goes da Silva, Emanuele Silva de Sousa, Bruno Bezerra da Silva, Lívia Maria Carlos Marques

**Affiliations:** 1 Universidade Estadual do Ceará, Avenida Paranjana, 1700 - Campus do Itaperi, Fortaleza - CE, 60740-000, Brazil; 2 GreenBean Biotechnology, Fortaleza, Brasil

## Background

Some plant viruses have been used to express and / or car peptides from human and animal pathogens for diagnostic purposes or vaccine. This system has presented many advantages over traditional methods, especially with regard to cost and production of peptides free of pathogens [1]. In this context, the possibility of using these viruses in an effort to produce peptide vaccine candidates in veterinary medicine is presented as a very promising idea. Among the relevant diseases in veterinary medicine there iscaprine artritre encephalitis (CAE) virus infection caprine arthritis-encephalitis (CAEV). This virus infects goats worldwide causing arthritis, encephalitis, mastitis, progressive weight loss and mainly fall in production and consequently a major economic loss to producers [2]. This disease is silent and yet there is still no treatment nor vaccine, and the control accomplished by early diagnosis. Therefore, this study aimed to produce peptides CAEV using a plant system.

## Methods

Primers were designed for a segment of p28 protein from the gag gene of CAEV. The insert obtained was cloned into pGEM, inserted into plasmid non-commercial (PNC) and inoculated on susceptible plants. The plants were kept in a greenhouse until the appearance of symptoms. The extraction of proteins was carried out [3] and the sample was submitted to SDS-PAGE and Western Blotting assay.

## Results and conclusions

The electrophoretic profile of approximately 55 kDa for the produced protein was similar to the positive control (commercial p28 protein). The Western Blotting confirmed the specific reactivity to this antigen. Thus, it was possible to determine the production of CAEV vaccine candidate peptides in plant systems.
